# Development of a Multiplex RT-qPCR for the Detection of Different Clades of Avian Influenza in Poultry

**DOI:** 10.3390/v12010100

**Published:** 2020-01-15

**Authors:** Tran Bac Le, Hye Kwon Kim, Woonsung Na, Van Phan Le, Min-Suk Song, Daesub Song, Dae Gwin Jeong, Sun-Woo Yoon

**Affiliations:** 1 Infectious Diseases Research Center, Korea Research Institute of Bioscience and Biotechnology, Daejeon 34141, Korea; letranbac86@kribb.re.kr; 2 Bio-Analytical Science Division, University of Science and Technology, Daejeon 34113, Korea; 3 Department of Microbiology, Chungbuk National University, Cheongju 28644, Korea; khk1329@chungbuk.ac.kr; 4 College of Veterinary Medicine, Chonnam National University, Gwangju 61186, Korea; wsungna@gmail.com; 5 College of Veterinary Medicine, Vietnam National University of Agriculture, Hanoi 100000, Vietnam; letranphan@gmail.com; 6 College of Medicine, Chungbuk National University, Cheongju 28644, Korea; songminsuk@chungbuk.ac.kr; 7 College of Pharmacy, Korea University, Sejong City 30019, Korea; songdaesop@gmail.com

**Keywords:** HPAI, H5Nx, Clade, simultaneous detection, field samples

## Abstract

Since the initial detection of H5N1, a highly pathogenic avian influenza (HPAI) virus, in 1996 in China, numerous HPAI H5 lineages have been classified, and they continue to pose a threat to animal and human health. In this study, we developed a novel primer/probe set that can be employed to simultaneously detect pan-H5 HPAI and two clades, 2.3.2.1 and 2.3.4.4, of H5Nx viruses using reverse transcription quantitative polymerase chain reaction (RT-qPCR). The sensitivity and specificity of these primer sets and probes were confirmed with a number of different subtypes of influenza virus and the H5-HA gene plasmid DNA. In particular, the multiplex RT-qPCR assay was successfully applied to the simultaneous detection of H5 HPAI and different virus clades in clinical field samples from a poultry farm. Therefore, this multiplex assay and a novel detection primer set and probes will be useful for the laboratory diagnosis and epidemiological field studies of different circulating H5 HPAI virus clades in poultry and migratory wild birds.

## 1. Introduction

Influenza A virus (IAV), a member of the genus *Orthomyxovirus*, causes a highly contagious respiratory disease in avian species and mammals [1]. Except for influenza H17 and H18 viruses that originated from bats, the waterfowl are known as a primordial reservoir for all subtypes of IAV [1,2]. Unlike human influenza viruses, avian influenza (AI) viruses are classified into two pathotypes—low pathogenic avian influenza (LPAI) and HPAI—based on the pathogenicity in chickens and molecular determinants of the multibasic cleavage site motif in the hemagglutinin (HA) protein [3,4]. During the circulation of HPAI virus subtypes H5Nx and H7Nx in poultry, they proved to be highly pathogenic with mortality of up to 100%. Sporadically, the H5N1 HPAI viruses cross the species barrier into humans. According to the WHO report [5], different subtypes of H5 HPAI viruses pose a substantial threat to global health with high mortality rate and huge economic burden to poultry farms.

Outbreaks of H5Nx HPAI viruses in poultry are increasing, and numerous clades of H5Nx HPAI have been classified since the first detection of the goose/Guangdong (Gs/GD) H5N1 HPAI virus in China [6]. However, the Gs/GD viruses and other IAVs have reassorted and diversified into 10 separate clades with more than 30 classified higher-order clades identified so far [7]. In particular, among the reassortment of novel H5Nx HPAI viruses, 2.3.2.1 and 2.3.4.4 clades of H5 HPAI viruses have become endemic in poultry populations in Asian countries, such as China, Vietnam, and South Korea [8,9]. The novel H5Nx viruses have reassorted with different NA subtypes, such as H5N2, H5N5, H5N6, and H5N8, in poultry and migratory birds [10]. The interaction between poultry and migratory wild birds and the complex overlapping flyways contribute to their long distance virus transmission [11]. The clade 2.3.2.1 of H5N1 HPAI virus was first identified in Hong Kong in 2004; since then other subclades, including 2.3.2.1a, 2.3.2.1b, and 2.3.2.1c, have evolved [12]. In addition, there was an outbreak of the H5 clade 2.3.4.4 virus around the East Asian-Australasian and American flyways in recent years [13,14,15]. Furthermore, these lineage viruses with different antigenic properties have been cocirculating. Thus, rapid and highly accurate methods are necessary for the detection of H5 HPAI viruses and clade identification.

Molecular diagnostic methods are used as the first step in the identification and control of the IAV outbreaks. Thus, a number of molecular methods, such as DNA microarrays [16], loop-mediated isothermal amplification tests [17], and restriction fragment mass analysis [18], have been developed and applied for rapid HA subtyping of the IAV field isolates [19]. Among these methods, RT-qPCR has been implemented in most reference diagnostics laboratories for the detection and characterization of IAVs. These diagnostic techniques were developed and used for subtyping or sequencing of novel viruses. However, due to the continuous circulation of H5Nx HPAI and other virus clades in avian species, it is very difficult to control viral transmission in poultry [3]. To effectively control and rapidly diagnose suspected infection on poultry farms, it is important to develop a rapid diagnostic assay for the simultaneous detection of different H5 HPAI viruses [20].

In this study, we tested our one-step multiplex method using clinical poultry samples to simultaneously detect pan-HPAI and two H5 clades. Thus, our one-step multiplex RT-qPCR assay can be used for the screening of virus-infected field samples.

## 2. Materials and Methods

### 2.1. Reference Viruses and Field Samples Collection

A total of 15 reference influenza virus samples were obtained from the Korea Research Institute of Bioscience and Biotechnology (KRIBB) and National Vietnam University of Agriculture. Coronavirus-229E and parainfluenza virus 1 were purchased from the Korea Bank for Pathogenic Viruses. Newcastle disease virus (VN1) and Infectious bronchitis virus (VNUA3) were kindly supported by Dr. Le Van Phan (National Vietnam University of Agriculture). All the reference influenza viruses were propagated in the allantoic fluid of 9–11 days old embryonated specific pathogen-free chicken eggs. All H5 HPAI experiments were conducted in a biosafety level 3 containment facility in KRIBB and biosafety level 2 plus facilities at the College of Veterinary Medicine, Vietnam National University of Agriculture, Hanoi, Vietnam. Field samples obtained from 12 farms in Vietnam were taken from domestic chickens with signs of respiratory illness in 2016 to 2018. For the screening of influenza positive field samples, all filed samples were analyzed using M-gene-specific RT-qPCR according to the WHO manual on animal influenza diagnosis and surveillance [21], and only M-gene positive field samples were used in the present study.

### 2.2. Primer and Probe Design

To detect the pan-H5 HPAI-specific and HPAI clade-specific probes, H5Nx HA gene sequences of 1018 low pathogenic and 4301 highly pathogenic avian influenza viruses from the GenBank database of the National Center for Biotechnology Information or Influenza Research Database (IRD, www.fludb.org) were analyzed, and complementary sequences were designed using ClustalW in BioEdit software (7.2 version) to identify conserved regions. The probes were labeled with the following fluorescent dyes: FAM for clade 2.3.2.1 detection, HEX for clade 2.3.4.4 detection, or Texas Red for H5 HPAI detection. The following primers and probes were used: HA forward primer, CCAGCCAATGACCTYTGT; HA reverse primer, GRTAAGCCAYACCACATTTC; Pan H5-HPAI probe, Texas red-TGARGAAYTGAAACACCTATTGAGCAG-BHQ2; Clade 2.3.2.1 probe, FAM-TCCCTGGTATGAACATGCTGCGCT-BHQ1; Clade 2.3.4.4 probe, HEX-TACCCAGGGAVCCTCAATGA-BHQ1. All amplicons obtained using one set of HA primers were 201 base pairs in length.

### 2.3. Sensitivity Analyses of One-Step Multiplex RT-qPCR Analysis

To assess the reaction efficiency and limit of detection of our multiplex qPCR assay, viral H5-HA gene was synthesized using the full-length H5-HA gene sequence from A/environment/Korea/W150/2006 for HPAI detection, A/Environment/Korea/W541/2016 for clade 2.3.4.4 detection, or A/wild bird/Jiangsu/H184/2015 for clade 2.3.2.1 detection and cloned using the Mighty TA cloning kit according to the manufacturer’s recommendations. After cloning, each H5-HA gene plasmid insertion was confirmed by DNA sequencing analysis. For the determination of limit of detection, one-step multiplex qPCR assays were performed using a serial 10-fold dilution (5 × 10^8^ to 5 × 10^0^ copies of plasmid DNA/μL) of each plasmid; each qPCR assay was performed in triplicate. The plasmid copy number was calculated using the URI Genomics & Sequencing Center program (http://cels.uri.edu/gsc/cndna.html). 

### 2.4. One-Step Multiplex RT-qPCR Analysis

For the one-step multiplex RT-qPCR, viral RNA was extracted using the RNeasy Mini Kit (Hilden, Germany) following the manufacturer’s instructions. The RT-qPCR assays were performed using the LightCycler 96 instrument and software (1.1 version). The one-step multiplex RT-qPCR reaction mixture contained 0.8 μL of enzyme mixture, 4 μL of 5× reaction buffer, 0.8 μL of deoxynucleoside triphosphate (10 pmol) from a Qiagen (Hilden, Germany) one-step RT-qPCR kit, 1 μL of each primer (10 pmol), 1 μL of each probe (5 μM), 3 μL RNA template and RNase-free water so that the final reaction volume reached 20 μL. The PCR thermocycling procedure was performed at 50 °C for 30 min and 94 °C for 10 min for the reverse transcription step; the next step was performed at 94 °C for 20 s, 56 °C for 20 s, and 72 °C for 30 s for 40 cycles. Fluorescence data were acquired during the annealing step to distinguish the positive and negative results. The RT-qPCR positive reaction of *c*t value was set to 35 and RNase-free water was used as a negative control. 

### 2.5. Specificity Analyses of One-Step Multiplex RT-qPCR Analysis

Specificity of our multiplex RT-qPCR system was evaluated with different influenza virus and other avian/human pathogens. total viral RNA samples from 1 human influenza virus (H1N1 subtype), 11 avian influenza viruses (H1N1, H3N3, H5N1, H5N2, H5N6, H5N8, H7N1, H9N2, and H10N1 subtype), 1 equine influenza virus (H3N8 subtype), 1 canine influenza virus (H3N2), 1 swine influenza virus (H3N2 subtype), avian infectious bronchitis virus, Newcastle disease virus, human Coronavirus-229E, and human Parainfluenza were assessed using one-step multiplex RT-qPCR described in this study. 

### 2.6. H5 HPAI Clade Assignment of Filed Samples

All H5 HPAI-positive field samples were identified by conventional reverse transcription polymerase chain reaction (RT-PCR), and the HA gene was amplified using the following primer set: forward 5′-GCATTGGYTAYCATGCAAAYA-3′ and reverse 5′-TTGCTRTGGTGRTAYCCATACA-3′. Specific amplicons were purified from 1.5% agarose gels using a QIAquick Gel Extraction Kit (Hilden, Germany) and sequenced using an ABI PRISM 3730 DNA Sequencer. The sequences were compiled and edited using the BioEdit program (www.mbio.ncsu.edu/BioEdit/bioedit.html). Based on their sequences, all H5 HPAI-positive field viruses were classified as the H5 HPAI clade using Highly Pathogenic H5N1 Clade Classification Tool in the Influenza Research Database.

## 3. Results

### 3.1. Sensitivity of One-Step Multiplex RT-qPCR Assay

**Figure 1:**
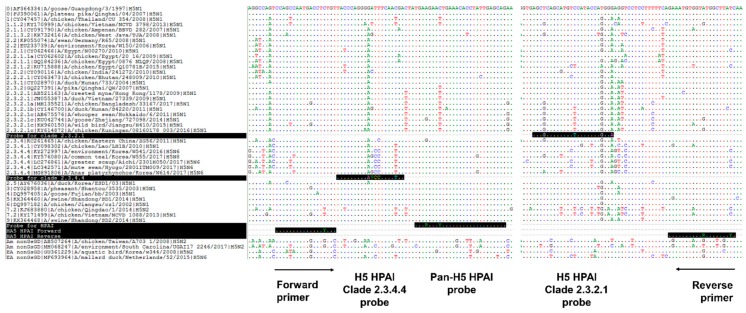
Design of the primer/probe set for the one-step multiplex RT-qPCR assay. Alignment of nucleotide sequences within the hemagglutinin (HA) gene of H5Nx avian influenza viruses that are representative of highly pathogenic avian influenza (HPAI) clades and low pathogenic avian influenza (LPAI) virus. The sequences of primers and probes specific to each target are on black background, and the reverse primer and probe specific for clade 2.3.2.1 show reverse complement sequence (R = A/G, Y = C/T, V = A/C/G).

**Figure 2:**
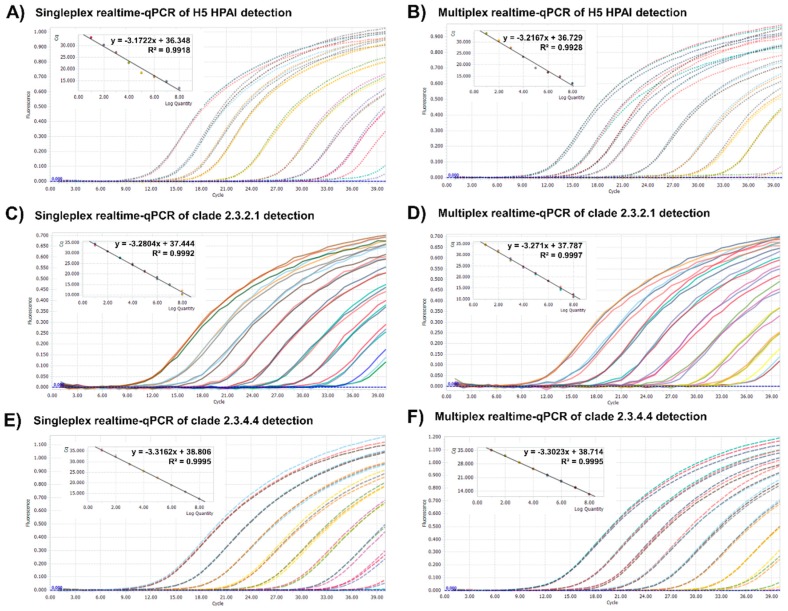
Amplification plots and standard curves of the singleplex and multiplex realtime-qPCR assays using target-specific plasmid DNA. Comparison of the singleplex (**A**,**C**,**E**) and multiplex (**B**,**D**,**F**) realtime-qPCR assays was performed using standard plasmids corresponding to H5 HPAI and clades 2.3.4.4 and 2.3.2.1; each plasmid solution was serially diluted down to 5 × 10^8^ to 5 × 10^0^ copies of plasmid DNA/microliter. The cycle threshold in amplification plots, correlation coefficient (R2) and slope of the standard curve for the assays were drawn automatically by Lightcycler 96 software. Each concentration had triple replicates.

To develop the one-step multiplex RT-qPCR assay, a specific primer set and the probes for pan-H5 HPAI and two clades, 2.3.4.4 or 2.3.2.1, were designed. In order to enhance the simultaneous detection specificity of a target gene, the H5 HPAI HA gene sequences from different years and various clades of H5 HPAI strains were downloaded from the GenBank database; primers and probes for the most conserved regions of the HA gene (Figure 1) were designed. To optimize our one-step multiplex qPCR, three HA gene plasmids, containing the H5 HPAI HA genes of two clades, were amplified and used as a quantitative standard in our system. Each plasmid was serially diluted (5 × 10^8^ to 5 × 10^0^ copies per microliter), and the sensitivity of our primer/probe set was determined using the singleplex and multiplex qPCR assays targeting pan-H5 HPAI, clade 2.3.4.4, or 2.3.2.1. The standard curves, generated for these targets under both qPCR conditions, showed linearity (*R*^2^ = 0.9918 to 0.9997, Figure 2). Also, we showed that the detection limit under both qPCR conditions was 5 × 10^1^ copies per microliter (Table 1). The cut-off for the cycle threshold of RT-qPCR was set to 35.

### 3.2. Specificity of One-Step Multiplex RT-qPCR Assay

To assess analytical specificity of the one-step multiplex RT-qPCR assay, strains of different subtypes or clades of influenza A virus, as well as other avian or human origin viruses, infectious bronchitis virus, Newcastle disease virus, coronavirus-229E strain, and parainfluenza virus 1, were tested. As we expected, all H5 HPAI and two clade viruses were successfully detected, while the other influenza A virus subtypes and viruses of other origin were not amplified by our multiplex RT-qPCR (Table 2). In addition, the H5 LPAI subtype viruses were not detected using our specific H5 HPAI primer/probe set. Interestingly, although the H5N1 HPAI subtypes of A/environment/Korea/W150/2006 (clade 2.2) and A/duck/Vietnam/NCVD-1648/2012 (clade 2.3.2.1) are the same, our primer/probe set detected only clade 2.3.2.1. These results show that our multiplex RT-qPCR assay is highly specific and exhibits no cross-reactivity for H5 HPAI and the other clades.

### 3.3. Application of One-Step Multiplex RT-qPCR Assay Using Clinical Samples

**Figure 3:**
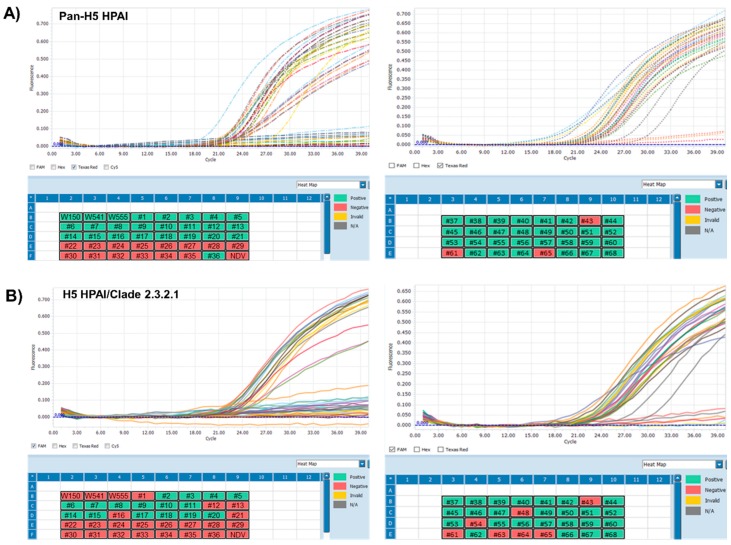

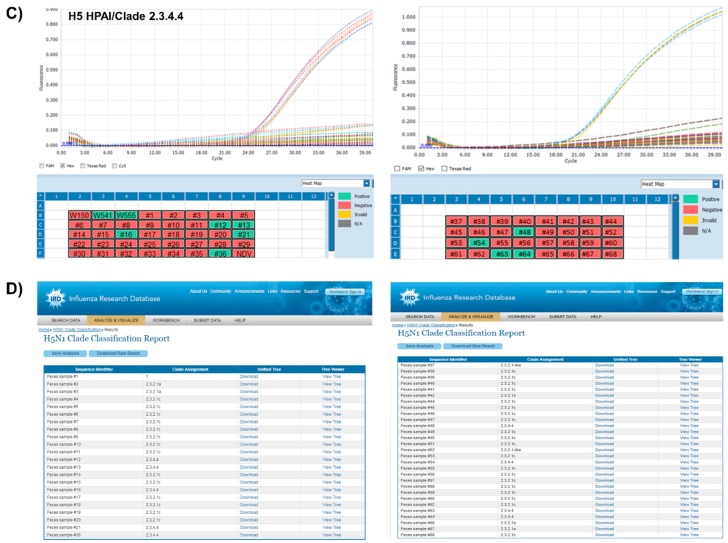
The one-step multiplex RT-qPCR assay for the detection of pan-H5 HPAI (**A**), H5 HPAI 2.3.2.1 clade (**B**), and H5 HPAI 2.3.4.4 clade (**C**) in field-infected feces samples. The validity of this assay was confirmed using M-gene positive field samples, H5Nx HPAI viruses for positive control, and Newcastle Disease virus for negative control. The results were visualized using an intuitive heat-map (green color-positive result, red color-negative result). Each concentration had triple replicates. (**D**) Clade classification of HPAI H5 subtype viruses from field-infected feces samples. The HA1 partial sequences of the HA gene with multiple basic cleavage sites were obtained by conventional RT-PCR and all H5 HPAI-positive sequences were verified using the Influenza Research Database (www.fludb.org).

To detect H5 HPAI in fecal samples using our one-step multiplex RT-qPCR assay, we obtained a total of 4350 fecal samples from domestic chickens with signs of respiratory illness in Vietnam, and viral RNA was isolated from these samples. A total of 68 samples were confirmed influenza positive by M-gene-specific RT-qPCR. Among the IAV-positive clinical samples, 51 samples were H5 HPAI-positive, and each clade was successfully analyzed using our one-step multiplex RT-qPCR assay (Table 3 and Figure 3). Next, we confirmed the sequence of all H5 HPAI-positive samples using the universal HA sequencing primers, forward 5′-GCATTGGYTAYCATGCAAAYA-3′ and reverse 5′-TTGCTRTGGTGRTAYCCATACA-3′, and classified each clade using the Influenza Research Database. Interestingly, when we have compared with sequencing analysis and clade identification using each amplicon, the primers and probes for the pan-H5 HPAI-specific and two clade-specific multiplex RT-qPCR assays were well-matched (Figure 3D). These results show that our one-step multiplex RT-qPCR assay could be a promising technique for specific discrimination of multiple H5 HPAI viruses in field samples from infected avian species. 

## 4. Discussion

Since H5N1 HPAI viruses was first identified in 1996, diverse subtypes of H5 HPAI were classified. Global and domestic spread of these virus caused huge losses to the poultry industry [18]. Novel H5Nx HPAI viruses appeared due to reassortment of the HA gene with different NA genes. According to the WHO report [22], H5Nx HPAI viruses have been classified into 13 clades based on their HA sequences. Recently, new clade classifications appeared (1.1.1, 7.1, 2.1.3.1, 2.1.3.3, 2.2.1.1, 2.2.1.1a, 2.2.2, 2.2.2.1, 2.3.2.1, 2.3.4.1, 2.3.4.3, and 2.3.4.4) owing to the emerging HPAI virus subtypes H5N2, H5N3, H5N5, H5N6, and H5N8 [18]. Among several new clades, two clades of H5 HPAI virus, 2.3.4.4 and 2.3.2.1, have been recently prevalent and dominant in Asia such as China [23], South Korea [24], and Vietnam [25]. In particular, 2.3.4.4 clade of H5 virus has spread to China and neighboring countries, such as South Korea [24], Vietnam [25], and Laos [26] between 2013 and 2016.

Despite many efforts to control the spread of H5 HPAI viruses [27] in poultry, such as development of a vaccine and diagnostic systems [28], viruses evolved and infected wild birds and domestic poultry. In particular, to effectively monitor and control H5Nx HPAI virus transmission, a number of diagnostic techniques, including virus isolation and conventional RT-qPCR, have been reported [29]. Virus isolation is the gold standard for influenza virus diagnosis, but it is time-consuming, expensive, and not suitable for discrimination of multiple HPAI viruses. The conventional RT-qPCR assay is well-known and useful for molecular detection of influenza viruses such as influenza virus subtyping and positive detection using matrix gene detection [30]. In particular, this method detects the fluorescence signal of a positive sample in real time due to using of the target-specific probe/primers, resulting in high sensitivity and specificity. Therefore, the RT-qPCR assay could be widely used as molecular diagnostic technology in clinical fields. To detect H5 HPAI virus and difference of H5 HPAI clade viruses in clinical samples, a multiplex RT-qPCR assay has been developed using several pairs of primers and probes [29,31,32]. However, some drawbacks are the reduced specificity and sensitivity of the target gene amplification due to the nonspecific interaction among primers and probes in the same reaction [32].

In this study, novel primers and probes were designed for the pan-H5 HPAI detection and the classification of two clades of H5 HPAI, 2.3.4.4 or 2.3.3.1, in field samples. Our one-step multiplex RT-qPCR assay was successfully employed to amplify viruses and IAV-positive field samples using novel primers/probes for the simultaneous detection of pan-H5 HPAI and two clades without cross-reactivity. To enhance the specificity of target detection, we designed a new single primer set and each probe for the conserved sequences of H5 HPAI. After testing the IAV-positive clinical field samples, 51 were H5 HPAI positive and the cycle threshold values of these positive samples ranged from around 20 to 24. Moreover, all H5 HPAI-positive samples were sequenced, and the results were verified by GenBank BLAST processing. Interestingly, the limit of detection of pan-H5 HPAI and clades in the singleplex and multiplex RT-qPCR assays was the same when our new single primer set and each probe combination was used.

## 5. Conclusions

Our one-step multiplex RT-qPCR method ensures highly specific and sensitive simultaneous detection of pan-H5 HPAI and two clades of H5 HPAI. Our method development focused on pan-H5 HPAI and two clades of H5 HPAI virus, 2.3.2.1 and 2.3.4.4, which have been dominant in Asia and present in Eurasia and Africa. This detection platform provides useful screening of clinical samples from domestic farms and live poultry markets.

## Figures and Tables

**Table 1:** Sensitivity of the single and multiplex assays.

Specificity	Fluorescence Dye	Singleplex Assay			Multiplex Assay		
		Limit of Detection	Mean *c*t Value	SD	Limit of Detection	Mean *c*t Value	SD
Clade 2.3.4.4	Hex	5 × 10^1^	34.39	0.025	5 × 10^1^	33.84	0.03
Clade2.3.2.1	FAM	5 × 10^1^	33.83	0.020	5 × 10^1^	34.5	0.025
Pan-H5 HPAI	Texas red	5 × 10^1^	34.12	0.061	5 × 10^1^	33.89	0.06

Each concentration had triple replicates and the mean and SD were conducted using GraphPad Prism software (version 5.1).

**Table 2:** Reference virus strains used for specificity analysis of the developed RT-qPCR.

Virus Name	Host	Accession Number	Subtype	Mean *c*t Value			Comments
				H5 HPAI	2.3.2.1c Clade	2.3.4.4 Clade	
A/California/04/2009	Human	GQ280797	H1N1	ND	ND	ND	
A/wild bird/Korea/SK14/2014	Avian	KX066871	H1N1	ND	ND	ND	LPAI
A/swine/Korea/CAN04/2005	Swine	EU798790	H3N2	ND	ND	ND	
A/canine/Korea/01/2007	Canine	JX163256	H3N2	ND	ND	ND	
A/aquatic bird/SouthKorea/sw006/2016	Avian	MG386182	H3N3	ND	ND	ND	LPAI
A/equine/Kyonggi/SA1/2011	Equine	JX844146	H3N8	ND	ND	ND	
A/environment/Korea/W150/2006	Avian	EU233739	H5N1	21.55	ND	ND	Clade 2.2
A/duck/Vietnam/NCVD-1648/2012	Avian	KY171342	H5N1	20.58	21.6	ND	Clade 2.3.2.1c
A/aquatic bird/Korea/CN2/2009	Avian	KY584075	H5N2	ND	ND	ND	LPAI
A/aquatic bird/ South Korea/sw007/2015	Avian	MG386197	H5N3	ND	ND	ND	LPAI
A/Environment/Korea/W541/2016	Avian	KY272997	H5N6	22.13	ND	21.36	Clade 2.3.4.4
A/Common Teal/Korea/W555/2017	Avian	KY576080	H5N8	20.82	ND	19.02	Clade 2.3.4.4
A/aquatic bird/ South Korea/sw001/2015	Avian	MF987893	H7N1	ND	ND	ND	LPAI
A/Chicken/Korea/MS96/1996	Avian	GU053186	H9N2	ND	ND	ND	LPAI
A/Aquatic bird/South Korea/SW1/2018	Avian	MK539837	H10N1	ND	ND	ND	LPAI
Infectious bronchitis virus	Avian	KY992863	VNUA3	ND	ND	ND	
Newcastle disease virus	Avian	KC607878	VN1	ND	ND	ND	
Coronavirus-229E, strain	Human	AY386391	KUMC-9	ND	ND	ND	
Parainfluenza virus 1	Human	MG255129	KUMC-44	ND	ND	ND	

ND: not detected. Each concentration had triple replicates.

**Table 3:** Application of the multiplex RT-qPCR to feces samples from field-infected chickens.

Samples Description	*c*t Value							Clade **
	IAV *	HPAI		2.3.2.1		2.3.4.4		
		Singleplex	Multiplex	Singleplex	Multiplex	Singleplex	Multiplex	
A/environment/Korea/W150/2006	+	20.32	20.04	ND	ND	ND	ND	2.2
A/Environment/Korea/W541/2016	+	20.68	21.33	ND	ND	21.32	22.09	2.3.4.4
A/Common Teal/Korea/W555/2017	+	19.79	21.81	ND	ND	21.62	22.01	2.3.4.4
Feces sample #1	+	25.35	26.3	ND	ND	ND	ND	1
Feces sample #2	+	21.82	21.29	23.72	24.07	ND	ND	2.3.2.1a
Feces sample #3	+	19.5	19.32	20.97	22.12	ND	ND	2.3.2.1a
Feces sample #4	+	19.84	19.72	20.05	20.93	ND	ND	2.3.2.1c
Feces sample #5	+	19.99	19.2	19.89	21.32	ND	ND	2.3.2.1c
Feces sample #6	+	21.37	21.53	22.18	21.9	ND	ND	2.3.2.1c
Feces sample #7	+	20.24	20.35	21.07	20.75	ND	ND	2.3.2.1c
Feces sample #8	+	19.88	19.57	19.93	20.49	ND	ND	2.3.2.1c
Feces sample #9	+	23.13	22.63	23.19	23.56	ND	ND	2.3.2.1c
Feces sample #10	+	22.29	22.15	22.64	23.26	ND	ND	2.3.2.1c
Feces sample #11	+	20.33	19.68	20.4	21.1	ND	ND	2.3.2.1c
Feces sample #12	+	20.13	21	ND	ND	20.7	21.97	2.3.4.4
Feces sample #13	+	19.48	22.59	ND	ND	22.33	21.24	2.3.4.4
Feces sample #14	+	20.2	19.97	20.54	20.45	ND	ND	2.3.2.1c
Feces sample #15	+	22.05	21.58	21.93	22.31	ND	ND	2.3.2.1c
Feces sample #16	+	21.64	23.66	ND	ND	23.54	23.45	2.3.4.4
Feces sample #17	+	20.85	20.56	20.88	21.44	ND	ND	2.3.2.1c
Feces sample #18	+	19.49	19.65	20.15	20.78	ND	ND	2.3.2.1c
Feces sample #19	+	20.92	21.02	21.6	21.98	ND	ND	2.3.2.1c
Feces sample #20	+	20.68	20.31	20.87	21.26	ND	ND	2.3.2.1c
Feces sample #21	+	22.04	23.54	ND	ND	23.15	23.13	2.3.4.4
Feces sample #22	+	ND	ND	ND	ND	ND	ND	LPAI
Feces sample #23	+	ND	ND	ND	ND	ND	ND	LPAI
Feces sample #24	+	ND	ND	ND	ND	ND	ND	LPAI
Feces sample #25	+	ND	ND	ND	ND	ND	ND	LPAI
Feces sample #26	+	ND	ND	ND	ND	ND	ND	LPAI
Feces sample #27	+	ND	ND	ND	ND	ND	ND	LPAI
Feces sample #28	+	ND	ND	ND	ND	ND	ND	LPAI
Feces sample #29	+	ND	ND	ND	ND	ND	ND	LPAI
Feces sample #30	+	ND	ND	ND	ND	ND	ND	LPAI
Feces sample #31	+	ND	ND	ND	ND	ND	ND	LPAI
Feces sample #32	+	ND	ND	ND	ND	ND	ND	LPAI
Feces sample #33	+	ND	ND	ND	ND	ND	ND	LPAI
Feces sample #34	+	ND	ND	ND	ND	ND	ND	LPAI
Feces sample #35	+	ND	ND	ND	ND	ND	ND	LPAI
Feces sample #36	+	23.21	24.33	ND	ND	23.86	23.92	2.3.4.4
Feces sample #37	+	26.71	27.4	27.01	27.48	ND	ND	2.3.2.1
Feces sample #38	+	20.49	21.48	21.01	20.98	ND	ND	2.3.2.1c
Feces sample #39	+	22.27	22.93	22.65	23.11	ND	ND	2.3.2.1c
Feces sample #40	+	21.89	22.36	21.82	22.56	ND	ND	2.3.2.1c
Feces sample #41	+	22.92	23.31	22.91	23.37	ND	ND	2.3.2.1c
Feces sample #42	+	19.46	19.62	19.38	19.96	ND	ND	2.3.2.1a
Feces sample #43	+	ND	ND	ND	ND	ND	ND	LPAI
Feces sample #44	+	23.17	23.71	23.16	24.16	ND	ND	2.3.2.1c
Feces sample #45	+	21.62	22.37	21.88	22.75	ND	ND	2.3.2.1c
Feces sample #46	+	29.58	29.98	29.41	30.42	ND	ND	2.3.2.1c
Feces sample #47	+	23.36	23.21	23.86	23.76	ND	ND	2.3.2.1c
Feces sample #48	+	18.88	18.94	ND	ND	19.88	20.05	2.3.4.4
Feces sample #49	+	23.6	24.3	23.42	24.52	ND	ND	2.3.2.1c
Feces sample #50	+	23.9	24.14	24.16	24.6	ND	ND	2.3.2.1c
Feces sample #51	+	22.32	22.85	22.71	23.22	ND	ND	2.3.2.1c
Feces sample #52	+	24.67	24.4	24.36	24.37	ND	ND	2.3.2.1c
Feces sample #53	+	21.32	22.06	21.22	21.71	ND	ND	2.3.2.1c
Feces sample #54	+	18.97	18.53	ND	ND	18.3	18.94	2.3.4.4
Feces sample #55	+	23.3	23.22	23.17	22.93	ND	ND	2.3.2.1c
Feces sample #56	+	22.42	22.79	22.94	23	ND	ND	2.3.2.1c
Feces sample #57	+	22.8	22.92	22.43	22.36	ND	ND	2.3.2.1c
Feces sample #58	+	25.22	25.04	24.17	25.05	ND	ND	2.3.2.1c
Feces sample #59	+	23.57	23.83	23.91	23.38	ND	ND	2.3.2.1c
Feces sample #60	+	23.06	23.78	23.54	23.39	ND	ND	2.3.2.1c
Feces sample #61	+	ND	ND	ND	ND	ND	ND	LPAI
Feces sample #62	+	24.81	24.75	23.93	24.55	ND	ND	2.3.2.1c
Feces sample #63	+	18.6	18.09	ND	ND	18.93	19.16	2.3.4.4
Feces sample #64	+	18.16	18.2	ND	ND	19.16	19.3	2.3.4.4
Feces sample #65	+	ND	ND	ND	ND	ND	ND	LPAI
Feces sample #66	+	23.79	23.94	24.28	24.4	ND	ND	2.3.2.1a
Feces sample #67	+	23.78	23.78	23.83	24.25	ND	ND	2.3.2.1a
Feces sample #68	+	24.25	24.37	24.47	24.66	ND	ND	2.3.2.1c

ND: not detected; LPAI: low-pathogenic avian influenza. * Primers and probes set for M gene specific performed using the protocol from the WHO manual on animal influenza diagnosis and surveillance [21]. + Influenza positive sample. ** All sequences were confirmed from GenBank at the National Center for Biotechnology Information (NCBI) or the Influenza Research Database (IRD, www.fludb.org). Each concentration had triple replicates.
